# Phase Compensation Sensor for Ranging Consistency in Inter-Satellite Links of Navigation Constellation

**DOI:** 10.3390/s17030461

**Published:** 2017-02-24

**Authors:** Zhijun Meng, Jun Yang, Xiye Guo, Mei Hu

**Affiliations:** College of Mechatronics Engineering and Automation, National University of Defense Technology, Changsha 410073, China; mzj727@126.com (Z.M.); john323@163.com (J.Y.); huluwa88@126.com (M.H.)

**Keywords:** inter-satellite link (ISL), navigation constellation, ranging consistency, phase compensation sensor

## Abstract

The performance of the global navigation satellite system (GNSS) can be enhanced significantly by introducing the inter-satellite links (ISL) of a navigation constellation. In particular, the improvement of the position, velocity, and time accuracy, and the realization of autonomous functions require the ISL distance measurement data as the original input. For building a high-performance ISL, the ranging consistency between navigation satellites becomes a crucial problem to be addressed. Considering the frequency aging drift and the relativistic effect of the navigation satellite, the frequency and phase adjustment (FPA) instructions for the 10.23 MHz must be injected from the ground station to ensure the time synchronization of the navigation constellation. Moreover, the uncertainty of the initial phase each time the onboard clock equipment boots also results in a pseudo-range offset. In this Ref., we focus on the influence of the frequency and phase characteristics of the onboard clock equipment on the ranging consistency of the ISL and propose a phase compensation sensor design method for the phase offset. The simulation and experimental results show that the proposed method not only realized a phase compensation for the pseudo-range jitter, but, when the 1 PPS (1 pulse per second) falls in the 10.23 MHz skip area, also overcomes the problem of compensating the ambiguous phase by directly tracking the 10.23 MHz to ensure consistency in the ranging.

## 1. Introduction

Position, velocity, and time (PVT) accuracy, integrity, continuity, and availability are the four major performance indicators for satellite navigation systems [1]; innovations and upgrades in navigation systems are constantly being undertaken to technically enhance these properties. The accuracy of the global navigation satellite system (GNSS) has currently been improved from several tens of meters to a meter-level. Typical technical measures adopted include the differential global positioning system (DGPS), represented by a wide area augmentation system (WAAS), for reducing or eliminating GPS measurement errors using the high correlation of the GPS error sources in time and space, thereby improving the user’s positioning accuracy to a meter or even a sub-meter level [2,3]; the integration of pseudolites with the GNSS improves the positioning geometric factors and coverage rate, enhancing the navigation and positioning accuracy [4,5]; usage of a three carrier ambiguity resolution (TCAR) technology and a binary offset carrier (BOC) signal system to improve the accuracy of the positioning solution [6,7,8,9], etc. Among the various techniques used to improve the GNSS performance, the inter-satellite link (ISL) technology has become a research hotspot in recent years. A National Aeronautics and Space Administration (NASA) senior engineer, K.P. Maine, who was responsible for the global position system (GPS) III ISL demonstration, has stated that the introduction of ISLs can significantly improve the accuracy of the ephemeris prediction, realize satellite autonomous integrity monitoring, increase the autonomous orbit determination, and improve the navigation system flexibility and expansibility [10]. The European Space Agency (ESA) has initiated several exploratory projects on the ISLs adopted in the Galileo system since 2007 [11]. Recently, with the rapid development of the BeiDou-2 program, ISLs have been used in the Beidou navigation system to achieve autonomous navigation [12]. The ISL has become a significant symbol of the new generation satellite navigation systems and its construction, an important consensus for several current GNSSs [13].

The accuracy of inter-satellite ranging is the key for determining the quality of the ISL construction and ranging consistency is a prerequisite for ranging accuracy. Ananda et al. set the precision of inter-satellite ranging to 0.5 m for simulating autonomous orbit determination [14]. When the range variance is 0.45 m and the international GNSS service (IGS) rapid ephemeris is used as the orbit initial value, the accuracy of the autonomous orbit determination in 180 days can be up to 6 m [15]. According to the present conclusion of the autonomous orbit determination and satellite-ground joint orbit determination, the accuracy of inter-satellite ranging should be at a decimeter-level. Owing to the frequency aging drift of the onboard clock and the relativistic effect of the navigation satellite [16], it is necessary to regularly adjust the phase and frequency of the onboard clock. Most of the signal generation and the time counts in the payload of a navigation satellite are directly or indirectly derived from a single 10.23 MHz clock; hence, the 10.23 MHz is adjusted. However, the work processing clock of the ISL payload (here after referred to as $f_{c}$) remains at the pre-adjusted state, causing a phase deviation between the 10.23 MHz and the $f_{c}$, and inducing an offset in the measurement pseudo-range. Moreover, the uncertainty of the initial phase between the 1 PPS (1 pulse per second) and the 10.23 MHz each time the onboard clock device boots also results in a pseudo-range offset, where 1 PPS is obtained by a count of the 10.23 MHz.

Currently, most research focusus on ISL signal system design, ISL signal dynamic characteristic analysis, ISL constellation design, and ISL extension applications. Reference [17] proposes a method that uses the navigation data to achieve a rapid acquisition of the ISL signal in highly dynamic conditions. Reference [18] suggests that changing the non-coherent integration to a coherent integration can increase the sensitivity of the ISL signal acquisition. A novel optimized model based on the shortest algorithm for an ISL navigation constellation is presented [19]. A new approach for determining the precise orbit and ephemeris of the GNSS using an ISL has been developed [20]. Reference [21] summarizes the ISL application method and analyzes the availability of space service for the ISL in navigation constellations, etc. However, the methods presented to date do not focus on the ranging consistency in the ISLs of a navigation constellation. Ranging consistency is affected by the onboard clock equipment, ISL payload device, space environment, and the source transmitter, etc. In this paper, we focus on the influence of the frequency and phase characteristics of the onboard clock equipment on the ranging consistency of an ISL and propose a phase compensation sensor design method for the phase offset of the onboard clock equipment.

The next section describes the ISL ranging principle and analyzes the questions regarding the ranging consistency by deviations in the phase and frequency. Section 3 presents the design approach for the sensor. The sensor phase detection error is analyzed in Section 4 and the sensor compensation performance is simulated and verified in Section 5. The final section presents our conclusions.

## 2. Ranging Principle and Analyses of the Questions

The dual-one-way measurement of an ISL consists of two satellites alternately performing a pseudo-code phase ranging process and the mutual transfer of the measurement results. It contains two one-way pseudo-range measurements.

**Figure 1:**
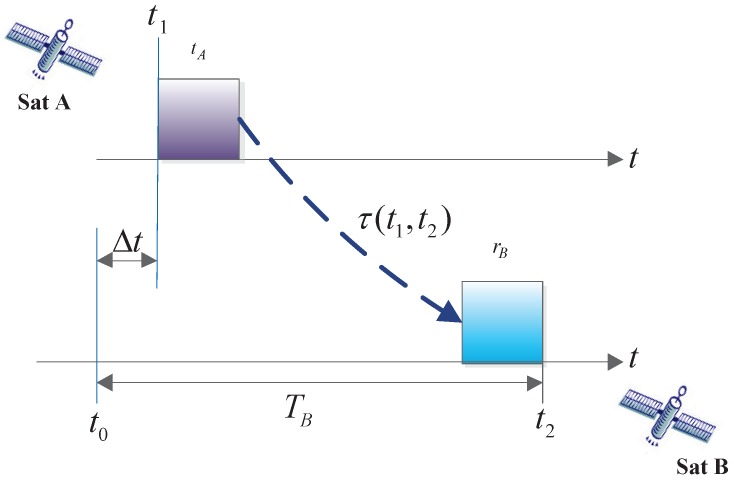
One-way pseudo-range measurement process.

As shown in Figure 1,

(1) $$T_{B}\;=\;\Delta t+t_{A}+\tau \left(t_{1},t_{2}\right)+r_{B}=t_{2}-t_{0},$$

where $T_{B}$ is the measurement pseudo-range, Δt is the system clock difference between satellite A and satellite B, $t_{A}$ is the transmitting circuit delay of satellite A, $r_{B}$ is the receiving circuit delay of satellite B, $\tau (t_{1},t_{2})$ is the spatial delay, $t_{1}$ is the transmitting point time of satellite A, and $t_{0}$ and $t_{2}$ are the reception point time and the measurement point time of satellite B, respectively. Next, we describe two questions in the development of the ISL payload engineering prototype.

### 2.1. Question 1

**Figure 2:**
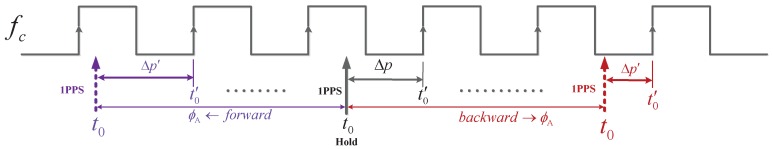
Uncertainty of the Δp between $t_{0}$ and ${t^{\prime }}_{0}$ with the FPA.

As shown in Figure 2, the satellite time, $t_{0}$, references the 1 PPS, which is the same as in Figure 1. In addition, ${t^{\prime }}_{0}$ is obtained by sampling the 1 PPS using the $f_{c}$. A phase difference, Δp, exists between $t_{0}$ and ${t^{\prime }}_{0}$. Furthermore, $t_{2}$ is also referenced to the 1 PPS and ${t^{\prime }}_{2}$ is obtained by a count of the $f_{c}$ to the measurement point time after the commencement of ${t^{\prime }}_{0}$. Likewise, a phase difference, Δp, also exists between $t_{2}$ and ${t^{\prime }}_{2}$.

In general, the 1 PPS is obtained by a count of the 10.23 MHz and $f_{c}$ is obtained by the frequency multiplication of the 10 MHz onboard atomic clock. If there is no frequency or phase skew, Δp remains fixed and can be calibrated in advance as the zero value of the equipment. However, with the FPA of the 10.23 MHz clock, a phase shift occurs between the 1 PPS and the $f_{c}$. When the phase shift is not an integer multiple of $f_{c}$, Δp changes. Assuming that the 10.23 MHz is shifted forward or backward by the phase, $\phi_{A}$, then $\Delta p^{\prime }$ denotes Δp after the phase shift. It can be expressed as:

(2) Δp′=remΔp+PA11fcfc,PA→forward,11fcfc−remΔp−PA11fcfc,PA→backward,

where $rem\left(•\right)$ represents the function of the remainder. When the frequency adjustment of the 10.23 MHz occurs, $\Delta p^{\prime }$ denotes Δp can be expressed as:

(3) Δp′=remΔp+ΔftΔft10.23e6+Δf10.23e6+Δf11fcfc,Δf≥0,11fcfc−remΔp−ΔftΔft10.23e6−Δf10.23e6−Δf11fcfc,Δf<0,

where Δf represents the frequency adjustment size and *t* is the time after the commencement of the adjustment. According to Equations (2) and (3), the phase uncertainty between $t_{0}$ and ${t^{\prime }}_{0}$ can be up to one working clock cycle. As per Equation (1), the ranging error is increased to an $f_{c}$ clock cycle. If the ISL payload working clock frequency is larger, the range error is smaller. However, considering the power consumption and the timing reliability of the processing chip, it is impossible to increase the working frequency by a considerable margin.

### 2.2. Question 2

After the onboard time-frequency equipment is powered on, it provides the ISL payload with the fixed phase relationship of the 1 PPS and the 10.23 MHz, and the phase relationship always remains at the boot state. However, the phase relationship of the 1 PPS and the 10.23 MHz is not the same each time the onboard time-frequency equipment boots.

**Figure 3:**
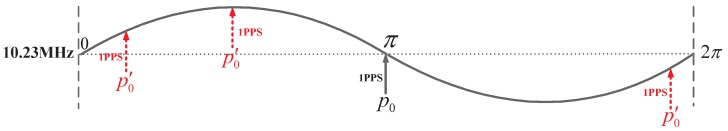
Uncertainty of the initial phase between the 1 PPS and the 10.23 MHz.

As shown in Figure 3, $p_{0}$ means that the system’s 1 PPS falls at the 10.23 MHz preset inital phase, ${p^{\prime }}_{0}$ means that the 1 PPS falls at the 10.23 MHz phase after each boot of the on-board time-frequency equipment. The 1 PPS may appear at any phase point of the 10.23 MHz. This uncertainty is a 10.23 MHz clock period of approximately 97.75 ns, resulting in an uncertainty of approximately 97.75 ns for $t_{0}$. This implies that the uncertainty of the ranging error caused by the onboard time-frequency equipment’s power switch is approximately 97.75 ns.

## 3. Proposed Sensor Design Method

Through the analyses of questions 1 and 2, we can see that the ranging deviations are related to the phase of the 10.23 MHz. Therefore, we must establish the phase relationship between $f_{c}$ and the 10.23 MHz. A design of the phase detection and compensation sensor based a digital phase-locked loop (PLL) for ranging deviations is herein presented.

According to literature [22], an *N* order phase-locked loop can accurately track signals, whose phases change with respect time to the power of N−1 or lower. Because the 10.23 MHz phase adjustment is equivalent to the phase step excitation, the frequency adjustment is equivalent to the frequency step excitation. Therefore, a second-order phase-locked loop can accurately track the phase changes after the FPA. The system function is [22]

(4) $$H\left(z\right)=\frac{T_{s}\left(b_{0}z^{-1}+b_{1}z^{-2}\right)}{{\left(1-z^{-1}\right)}^{2}+T_{s}\left(b_{0}z^{-1}+b_{1}z^{-2}\right)},$$

where

(5) $$\left\{\begin{array}{c} b_{0}=2\xi \omega_{n}+\frac{T_{s}}{2}{\omega_{n}}^{2},\\ b_{1}=-2\xi \omega_{n}+\frac{T_{s}}{2}{\omega_{n}}^{2}. \end{array}\right.$$

Here, $T_{s}$ is the data rate input to the loop filter, $b_{0}$ and $b_{1}$ are the loop filter parameters, $\omega_{n}$ is the system natural frequency, and *ξ* is the damping coefficient. With the 10.23 MHz phase-locked loop, we can accurately locate the 10.23 MHz phase at the sampling time, ${t^{\prime }}_{2}$. Assuming that the system’s 1 PPS falls at the 10.23 MHz phase, $p_{0}$ and at a phase, ${p^{\prime }}_{0}$, after a time-frequency equipment boot. It falls at a phase, $p_{1}$, through the ISL payload $f_{c}$’s sampling, and $p_{0}$, ${p^{\prime }}_{0}$, and $p_{1}$ are expressed as digital phases with bit widths of *N*. We then obtain:

(6) $$p_{compensate}=p_{1}-p_{0},$$

where $p_{1}$ is

(7) $$p_{1}=p_{0}^{\prime }+\Delta p.$$

**Figure 4:**
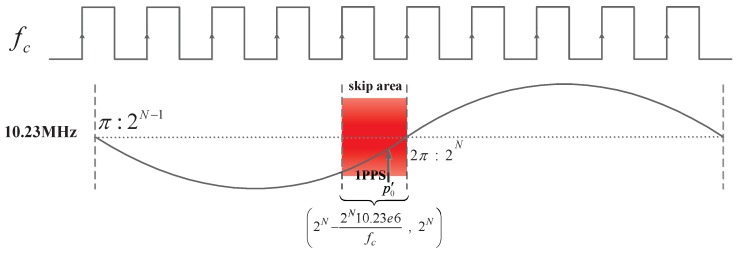
Skip area of the 10.23 MHz.

If the 1 PPS falls in the 10.23 MHz skip area, i.e., the region of one $f_{c}$ clock cycle (because the indefinite range of Δp is an $f_{c}$ clock cycle) to the left of the 2π (digital phase: $2^{N}$) phase of the 10.23 MHz, as shown in Figure 4 and if the 10.23 MHz has a phase deviation from $f_{c}$, the phase sampled at the rising edge of $f_{c}$ will be greater than $2^{N}$. However, when the sum of the digital phase accumulator exceeds $2^{N}$, it overflows and then accumulates from zero.

Therefore, if the 10.23 MHz is tracked by the PLL directly, the clock phase, $p_{1}$, of the 10.23 MHz at the sampling time, ${t^{\prime }}_{2}$, is

(8) $$\begin{array}{c} p_{1}=rem\left(\frac{{p^{\prime }}_{0}+\Delta p}{2^{N}}\right)\\ \;\;\;\;=\left\{\begin{array}{c} {p^{\prime }}_{0}+\Delta p,\;\;\;{p^{\prime }}_{0}+\Delta p<2^{N},\\ {p^{\prime }}_{0}+\Delta p-2^{N},\;\;\;{p^{\prime }}_{0}+\Delta p\geq 2^{N}. \end{array}\right. \end{array}$$

With the FPA of the 10.23 MHz, Δp has a phase uncertainty of 2N10.23e62N10.23e6fcfc. According to Equation (8), when ${p^{\prime }}_{0}+\Delta p$ is greater than $2^{N}$, the locked phase, $p_{1}$, output at time ${t^{\prime }}_{2}$ will move up and down around the $2^{N}$ phase of the 10.23 MHz. As per Equation (6), $p_{compensate}$ will thus have a phase error of $2^{N}$, namely, a 10.23 MHz clock period.

To avoid this error, we first divide the 10.23 MHz by *n* to obtain a frequency, $f_{n}$, by a direct digital synthesizer (DDS), yielding

(9) $$f_{n}=\frac{10.23e6}{n}.$$

Thus, the phase change of the 10.23 MHz is *n* times the phase change of $f_{n}$, which is,

(10) $$\phi_{f_{n}}=\frac{\phi_{10.23MHz}}{n}.$$

**Figure 5:**
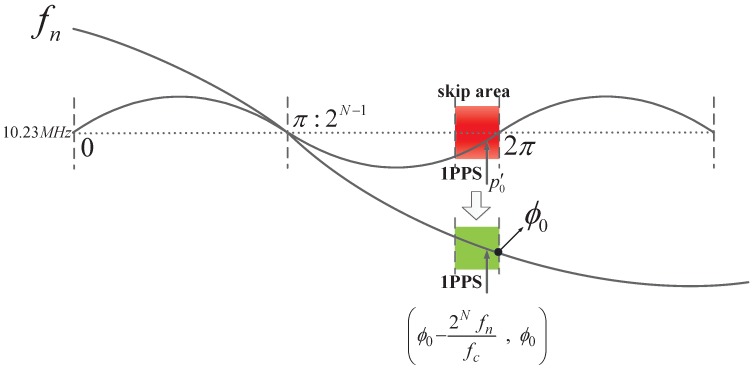
Transferring the skip area of the 10.23 MHz by $f_{n}$.

Then, we preset the initial phase, $\phi_{0}$, between the 10.23 MHz and $f_{n}$ by the DDS, whose activation must be triggered by the 1 PPS or any other signal directly associated with the 1 PPS. According to Figure 4, to ensure that the 1 PPS does not fall in the skip area of $f_{n}$, the lower boundary of $\phi_{0}$ must be greater than a 10.23 MHz clock period and the upper bound must be less than $2^{N}$ minus an $f_{c}$ clock period. We can then obtain $\phi_{0}\in \left(\frac{2^{N}}{n},2^{N}-\frac{2^{N}f_{n}}{f_{c}}\right)$. After this approach, as per Equation (10), the skip area in Figure 4 becomes $\left(\phi_{0}-\frac{2^{N}f_{n}}{f_{c}},\phi_{0}\right)$, as shown in Figure 5, where the value of $\phi_{0}$ is $2^{N-1}+\frac{2^{N-1}}{n}$.

Then, we make $f_{c}$ track $f_{n}$ using the PLL. At the sampling time ${t^{\prime }}_{2}$, it yields

(11) $$p_{1}=rem\left(\frac{{p^{\prime }}_{0}+\Delta p}{n2^{N}}\right).$$

Thus, as per Equation (11), as long as *n* is greater than one, $\phi_{0}$ will be greater than $\frac{2^{N}-{p^{\prime }}_{0}-\Delta p}{n}$ and a phase skip problem does not occur. Then, $p_{compensate}$ can be expressed as:

(12) $$p_{compensate}=n\cdot \left(p_{1}-p_{0}\right).$$

**Figure 6:**
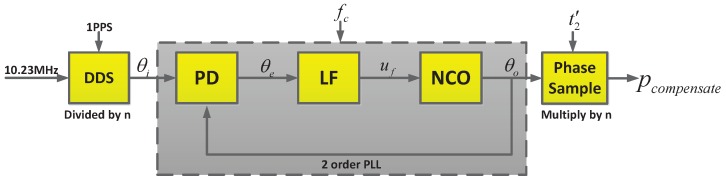
Entire process flow of the phase compensation.

The entire process flow of sensor is depicted in Figure 6.

Through phase compensation and the conversion of $p_{compensate}$ to time, the revised pseudo-range ranging formula is

(13) $$\begin{array}{c} T_{B}=\Delta t+t_{A}+\tau \left(t_{1},t_{2}\right)+r_{B}\\ \;\;\;=t_{2}-t_{0}+\frac{p_{compensate}}{2^{N}10.23e6}\\ \;\;\;=t_{2}-t_{0}+\frac{n\left(p_{1}-p_{0}\right)}{2^{N}10.23e6}, \end{array}$$

where *N* represents the bit width of the numerically controlled oscillator (NCO) of the 10.23 MHz phase.

## 4. Phase Error Analysis

In this Ref., the phase error of the sensor is mainly derived from the thermal noise of the phase-locked loop and the phase jitter noise of the onboard atomic clock. The estimation formula for the thermal noise mean square error, $\sigma_{t}$, is [22,23]

(14) σt=T02πBLCCN0N01+12TIntegral·CCN0N0,

where $B_{L}$ is the loop noise bandwidth:

(15) $$B_{L}=\frac{\omega_{n}}{2}\left(\xi +\frac{1}{4\xi }\right).$$

$C/N_{0}\;$ is the carrier-to-noise ratio of the input signal, $T_{0}$ is the input carrier period, and $T_{Integral}$ is the loop coherent integration time. The phase jitter noise, $\sigma_{A}$, of the onboard clock is proportional to the Allan Deviation mean square error, $\sigma_{A}\left(\tau \right)$ and the coherent integration time, $T_{Integral}$

(16) $$\sigma_{A}=T_{Integral}\sigma_{A}\left(\tau \right).$$

Based on the above two partial error sources, the total phase jitter mean square error is

(17) $$\sigma_{total}=\sqrt{{\sigma_{t}}^{2}+{\sigma_{A}}^{2}}.$$

Under normal circumstances, the onboard clock of the navigation satellite’s $C/N_{0}\;$ is better than 120 dB·Hz (10${}^{12}$ Hz) and $\sigma_{A}\left(\tau \right)$ is $10^{-12}$. Consider $B_{L}$ as 20 Hz (we set the classic parameters $\omega_{n}$ = 37.7 and *ξ* = 0.7) and $T_{Integral}$ is 1 ms. According to Equations (14), (16), and (17), $\sigma_{total}$ is approximately equal to 0.0696 ps. Set *n* equal to 4; as per Equation (12), the error of $p_{compensate}$ is 4$\sigma_{total}$, namely, 0.278 ps, that is considerably lesser than the accuracy of the 0.5 ns pseudo-code ranging [24] and a 10.23 MHz clock cycle.

## 5. Experimental and Simulation Results

**Figure 7:**
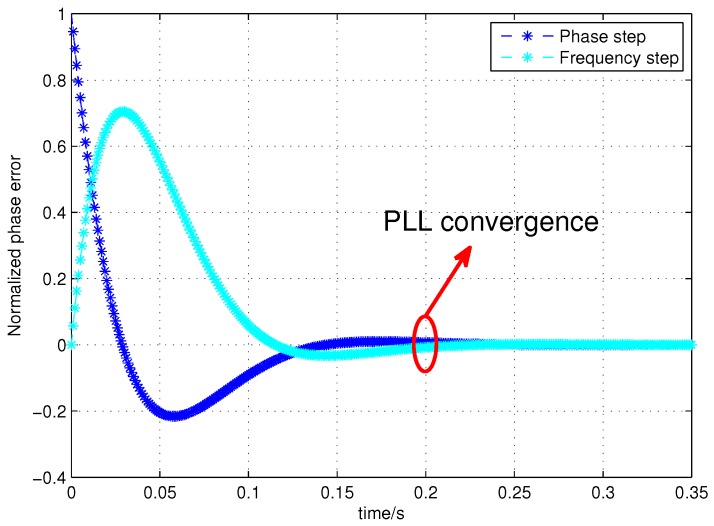
Error response of the frequency and phase step excitation.

Assuming that the one-way measurement period of the ISL is 1.5 s (GPS assigns 1.5 s to each satellite in ISLs) [14]. The clock, $f_{c}$, is 50 MHz and the system initial phase, $p_{0}$, is $2^{N-1}$ (namely, *π*). Assuming that the two satellites remain stationary, Δt can be monitored in real-time; the inter-satellite pseudo-range delay is 78 ms, the pseudo-code ranging accuracy of the ISL is 0.5 ns, the coefficients $\omega_{n}$ and *ξ* of the PLL’s system function are 37.3 and 0.7, respectively, and the data update rate, $T_{s}$, is 1 ms. Then, the response of the second-order PLL error system function under a frequency and phase step excitation is shown in the Figure 7.

**Figure 8:**
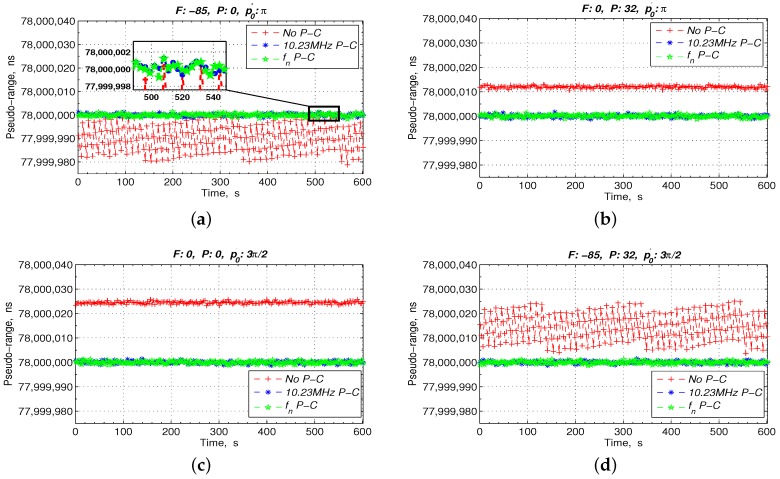
Ranging results with the 1 PPS in the non-skip-area. (**a**) only frequency adjustment; (**b**) only phase adjustment; (**c**) only change $p_{0}^{\prime }$; (**d**) the FPA and the changed $p_{0}^{\prime }$.

**Figure 9:**
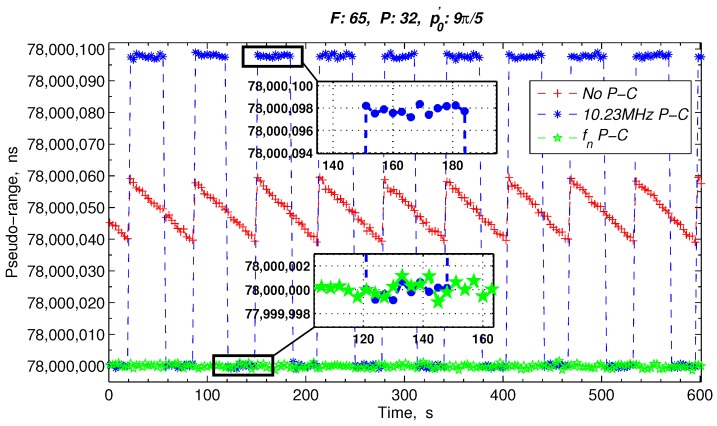
Ranging results with the 1 PPS in the skip-area.

Assuming that the DDS frequency division coefficient, *n*, is 4, the initial phase, $\phi_{0}$, is $2^{N-1}+\frac{2^{N-1}}{n}$ and the bit width of the phase accumulator, *N*, is 32. Then, the phase compensation of the ranging results in case of the FPA of the 10.23 MHz and the change in the ${p^{\prime }}_{0}$ of the 1 PPS are simulated. When the 1 PPS is in the non-skip-area, the pseudo-range measurement results with and without the phase compensation are shown in Figure 8. When the 1 PPS is in the skip area, the pseudo-range measurement results are shown in Figure 9. Table 1 lists the expansion of the abbreviations in Figure 8 and Figure 9, and the FPA values of the 10.23 MHz and the initial phase values of the 1 PPS are listed in the headers of Figure 8 and Figure 9.

As can be seen from Figure 7, the PLL only needs 200 ms to output the convergent locked phase after the FPA, which can guarantee the validity of the phase compensation data in a one-way measurement period (1.5 s).

From Figure 8, if there is no phase compensation, the pseudo-range values have an approximately 20 ns periodic jitter caused by the frequency adjustment as shown in Figure 8a, an approximately 12 ns fixed offset caused by the phase adjustment as shown in Figure 8b, an approximately 24.43 ns fixed offset caused by the change in the ${p^{\prime }}_{0}$ of the 1 PPS depicted in Figure 8c, and a 24.43 ns fixed offset and a 20 ns periodic jitter caused by the FPA and the changed ${p^{\prime }}_{0}$ as shown in Figure 8d. However, the ranging results with a phase compensation by tracking the 10.23 MHz directly or by tracking the $f_{n}$ are maintained at approximately 78 ms in Figure 8a–d.

Particularly, in Figure 9, when the 1 PPS is in the skip area, the measurement pseudo-range has an approximately 97.75 ns periodic jump with a phase compensation by directly tracking the 10.23 MHz. Nonetheless, after the proposed phase compensation sensor, the inter-satellite measurement results are maintained at approximately 78 ms.

**Figure 10:**
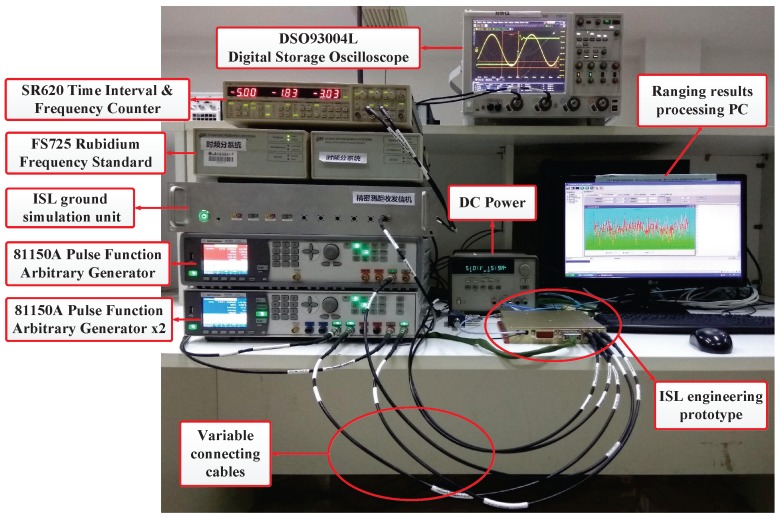
Experimental scenario of field data collection and ranging comparison.

To verify that the proposed phase compensation method is valid, we used an ISL engineering prototype for a field data acquisition and ranging comparison. We connected by coaxial cables the ISL ground simulation unit to the ISL engineering prototype via the intermediate frequency signal. The phase compensation sensor was realized by the field programmable gate array (FPGA) inside the prototype. The PLL setting parameters were the same as the simulation parameters. The experimental scenario is shown in Figure 10.

A single Stanford FS725 Rubidium Frequency Standard provided the ISL ground simulation unit with the 10 MHz and 1 PPS reference. The ISL engineering prototype 10 MHz reference was provided by another Stanford FS725. The latter one additionally provided the 10 MHz reference for two Agilent 81150A pulse function arbitrary generators (Agilent, Kuala Lumpur, Malaysia) The ISL prototype had a 50 MHz work clock, $f_{c}$, from a single-channel 81150A . It obtained the 1 PPS and the 10.23 MHz input from a dual-channel 81150A. The frequency adjustment was realized by controlling the output frequency of the dual-channel 81150A 10.23 MHz and 1 PPS. Meanwhile, the phase adjustment was realized by simultaneously changing the cable length of the 10.23 MHz. Furthermore, the uncertainty of the initial phase, $p_{0}^{\prime }$, was realized by repeatedly switching on the FS725 and measured by the Agilent DSO93004L digital storage oscilloscope.

**Figure 11:**
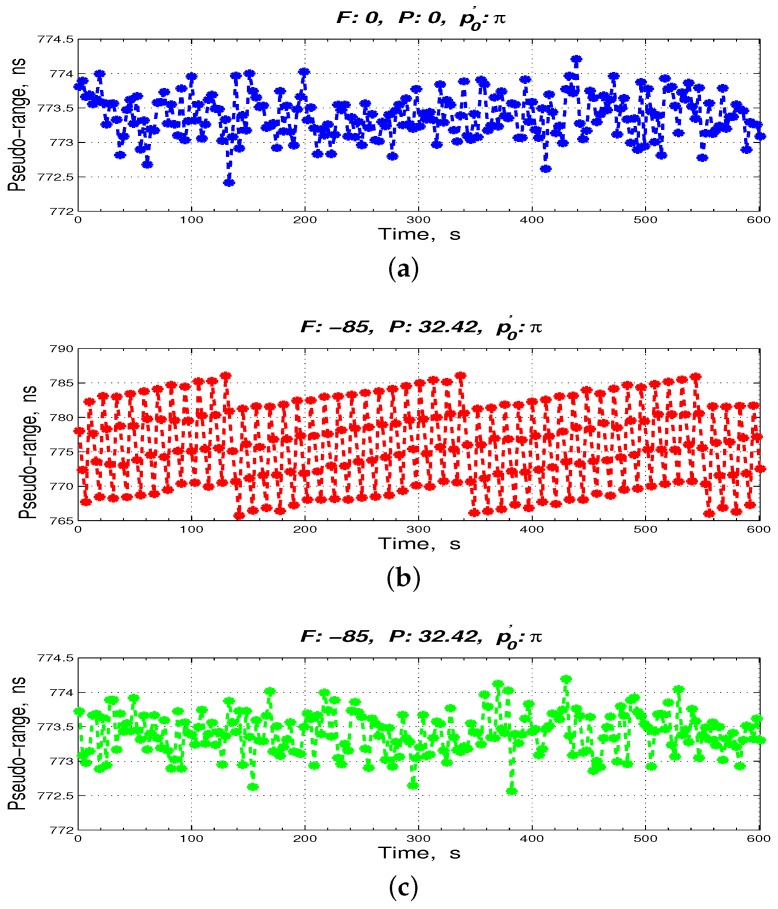
Collected actual comparison ranging data. (**a**) non-execution of the FPA without the phase compensation sensor; (**b**) execution of the FPA without the phase compensation sensor; (**c**) execution of the FPA with the phase compensation sensor.

In addition, DC power was supplied to the ISL prototype. The ranging results processing personal computer (PC) (Lenovo, Beijing, China) handled the prototype work instructions and measurement data acquisition. The Stanford SR620 time interval and frequency counter monitored the real-time clock error, Δt, and output it to the ranging results processing PC. The initial connection state shown in Figure 10 was deemed an unexecuted the FPA state; its ranging value is shown in Figure 11a. We simultaneously extended the cable by 8 m (32.42 ns) to the 1 PPS and the 10.23 MHz of the ISL prototype and decreased the 10.23 MHz by 85 mHz. The pseudo-range measurement results with and without the phase compensation sensor are shown in Figure 11b,c, respectively.

As shown in Figure 11, the measured ranging data are in accordance with the simulation results. For question 2, we powered off and on the FS725 of the ISL prototype five times without the phase compensation sensor, as well as with the phase compensation sensor. Then, we collected the ranging data, as shown in Table 2 and Table 3, respectively. The phase of the 1 PPS at the clock 10.23 MHz, $p_{0}^{\prime }$, could be read by the digital storage oscilloscope DSO93004L.

As per Table 2 and Table 3, after phase compensation sensor, the five times of ranging results were maintained at approximately 773.385 ns, which ensured the consistency of ISL ranging.

## 6. Conclusions

This paper first analyzes the inter-satellite pseudo-range jitter caused by the FPA of the navigation reference frequency of 10.23 MHz and the uncertainty of the initial phase between the 1 PPS and the 10.23 MHz. It then proposes a real-time phase compensation sensor design method based on the integer-n frequency of the second-order phase-locked loop. Through simulation analysis and experimental verification, the designed sensor’s phase detection error can reach the picosecond level, and the proposed phase compensation method not only realizes a phase compensation for the pseudo-range jitter caused by the FPA of the 10.23 MHz and the uncertainty of the initial phase between the 1 PPS and the 10.23 MHz, but also when the 1 PPS falls in the 10.23 MHz skip area, overcomes the problem of compensating the ambiguous phase by directly tracking the 10.23 MHz, guaranteeing the consistency of the ISL ranging.

In the near future, ISLs will be widely applied in all aspects of space services and will become another fundamental resource for navigation and communication satellites. To further improve the ranging consistency, accuracy, and applications of ISLs, we plan to consider incorporating ISL payload devices, space environments, and source transmitters in our analysis and design. We will also consider different constellations with different ISL ranging features rather than limiting our work to the navigation constellation.

## Figures and Tables

**Table 1:** Expansion of the abbreviation in Figure 8 and Figure 9.

Abbreviations	Definition
*F*	Frequency adjustment of the 10.23 MHz, unit: mHz
*P*	Phase adjustment of the 10.23 MHz, unit: ns
$p_{0}^{\prime }$	Initial phase of the 1 PPS, unit: rad
No P-C	Ranging results without phase compensation
10.23 MHz P-C	Phase compensation by tracking the 10.23 MHz
$f_{n}$ P-C	phase compensation by tracking $f_{n}$

**Table 2:** Collected actual power switch ranging data without the phase compensation sensor.

Switch Number	Ranging Value, unit: ns	$p_{0}^{\prime }$, unit: rad
1	755.399	0.632π
2	781.742	1.171π
3	787.317	1.285π
4	736.970	0.255π
5	803.395	1.614π

**Table 3:** Collected actual power switch ranging data with the phase compensation sensor.

Switch Number	Ranging Value, unit: ns	$p_{0}^{\prime }$, unit: rad
1	773.386	1.431π
2	773.389	0.762π
3	773.390	0.897π
4	773.381	1.833π
5	773.385	1.128π

## References

[1] Kovach K. Continuity: The hardest GNSS requirement of all. Proceedings of the Proceedings of the 11th International Technical Meeting of the Satellite Division of The Institute of Navigation (ION-GPS-98).

[2] Witte T.H., Wilson A.M. (2005). Accuracy of WAAS-enabled GPS for the determination of position and speed over ground. J. Biomech..

[3] Wang G.Y., Guo B.Y. (1996). Differential GPS Positioning Technique and Its Application.

[4] Klein D., Parkinson B.W. (1986). The use of pseudolites for improving GPS performance. Navigation.

[5] Xu H.L. (2003). Research on GNSS Performance Enhancement Technology.

[6] Tan S.S., Zhou B., Guo S.T. Design of global satellite navigation signal. Proceedings of the 1st China Satellite Navigation Conference.

[7] Navstar GPS Joint Program Office (2012). IS-GPS-200F: Navstar GPS Space Segment/navigation Use Interfaces. http://www.gps.gov/.

[8] European Union (2011). Galileo Signal in Space Interface Control Document (Issue 1.1). http://ec.europa.eu/.

[9] Russian Institute of Space Device Engineering (2008). GLONASS Interface Control Document (Edition5.1). http://www.novatel.com/.

[10] Maine K.P., Anderson P., Langer J. Crosslinks for the next-generation GPS. Proceedings of the Aerospace Conference.

[11] Amarillo F., Gerner J.L., Sanchez M. The ESA GNSS+ project: Inter-Satellite ranging and communication links in the frame of the GNSS infrastructure evolutions. Proceedings of the 21st International Technical Meeting of the Satellite Division of the Institute of Navigation.

[12] Liu J., Geng T., Zhao Q. (2011). Enhancing precise orbit determination of Compass with inter-satellite observations. Surv. Rev..

[13] Tan S.S. (2008). The development and consideration of Beidou navigation satellite system. J. Astronaut..

[14] Ananda M.P., Bernstein H., Cunningham K.E., Feess W.A., Stroud E.G. Global positioning system (GPS) autonomous navigation. Proceedings of the 1990 IEEE Position Location and Navigation Symposium.

[15] Zeng X.P. (2004). Navigation Satellite Autonomous Orbit Determination and Simulation Resuilts.

[16] Pogge R. (2014). Real-World Relativity: The GPS Navigation System.

[17] Li X.B., Wang Y.K., Chen J.Y. (2014). Rapid acquisition assisted by navigation data for inter-satellite links of navigation constellation. IEICE Trans. Commun..

[18] Tang Y.Y., Wang Y.K., Chen J.Y., Guo X.Y. (2015). High-sensitive acquisition of signals for inter-satellite links of navigation constellation. Electron. Lett..

[19] Yang Z.Q., Ji R., Lu Z.P., Shen Y., Shao S. Analysis and Design of An Optimized Constellation Inter-Satellite. Proceedings of the 2014 IEEE Chinese Guidance, Navigation and Control Conference.

[20] Wolf R. Satellite Rrbit and Ephemeris Determination Using Inter Satellite Links. https://www.cs.tcd.ie/Stephen.Farrell/ipn/background/orbit-calc-using-intersat-links.pdf.

[21] Tang Y.Y., Wang Y.K., Chen J.Y. (2016). The Availability of Space Service for Inter-Satellite Links in Navigation Constellations. Sensors.

[22] Kaplan E. (2006). Understanding GPS: Principles and Application.

[23] Parkinson B., Spilker J., Axelrad P. (1996). Global Positioning System: Theory and Applications.

[24] Holmes J.K., Raghavan S. A summary of the new GPS IIR-M and IIF modernization signals. Proceedings of the 2004 IEEE 60th Vehicular Technology Conference (VTC2004-Fall).

